# Towards universal health coverage: a mixed-method study mapping the development of the faith-based non-profit sector in the Ghanaian health system

**DOI:** 10.1186/s12939-018-0810-4

**Published:** 2018-10-05

**Authors:** Annabel Grieve, Jill Olivier

**Affiliations:** School of Public Health and Family Medicine, University of Cape Town, Cape Town, South Africa

**Keywords:** Faith-based providers, Ghana, Universal health coverage, GIS, Map, Public-private partnership, Non-state providers, Health system, History

## Abstract

**Background:**

Faith-based non-profit (FBNP) providers have had a long-standing role as non-state, non-profit providers in the Ghanaian health system. They have historically been considered to be important in addressing the inequitable geographical distribution of health services and towards the achievement of universal health coverage (UHC), but in changing contexts, this contribution is being questioned. However, any assessment of contribution is hampered by the lack of basic information about their comparative presence and coverage in the Ghanaian health system. In response, since the 1950s, there have been repeated calls for the ‘mapping’ of faith-based health assets.

**Methods:**

A historically-focused mixed-methods study was conducted, collecting qualitative and quantitative data and combining geospatial mapping with varied documentary resources (secondary and primary, current and archival). Geospatial maps were developed, providing a visual representation of changes in the spatial footprint of the Ghanaian FBNP health sector.

**Results:**

The geospatial maps show that FBNPs were originally located in rural remote areas of the country but that this service footprint has evolved over time, in line with changing social, political and economic contexts.

**Conclusion:**

FBNPs have had a long-standing role in the provision of health services and remain a valuable asset within national health systems in Ghana and sub-Saharan Africa more broadly. Collaboration between the public sector and such non-state providers, drawing on the comparative strengths and resources of FBNPs and focusing on whole system strengthening, is essential for the achievement of UHC.

## Background

Health systems are defined by the World Health Organisation (WHO) as “all organisations, people and actions whose primary intent is to promote, restore or maintain health” [1]. Present-day systems are the result of decades of change, having been strongly shaped by historical social, political and economic events [2]. As inherently social institutions, people are the central component of health systems with changing relationships and power dynamics between the various actors (both within and outside the system) also strongly influencing their composition [3]. A historical lens provides the opportunity to examine why a health system has evolved to its current state, what factors have been important in change [4], how to predict and possibly prevent similar (unintended) consequences in the future and what this might mean for better understanding the complex adaptations of the current system. Some of these system changes are reflected in and can be traced through the physical representation and geographic distribution of health infrastructure.

In many low- and middle-income countries (LMICs), the continued inequitable geographical distribution of the health system is a significant hindrance to the achievement of universal health coverage (UHC) and the goal of quality accessible services for all [5]. The concept of access is multifaceted but physical location is still fundamental in LMICs, influencing aspects of utilisation and quality and is frequently referred to as a continued significant barrier to those seeking and utilising services (to access) [6–8].

In order to address these ongoing public health challenges, there has been increasing global recognition of the necessity and value of leveraging private or non-state providers (NSPs) 1 that already constitute part of LMIC national health systems [9–11]. In many sub-Saharan African (SSA) countries, significant amongst these NSPs are *faith-based non-profit providers* (FBNP)^2^ who have had a longstanding role in service delivery and the achievement of public health goals (present pre-independence in most countries). The form they take and relationship they have with the government varies [12] and has changed over time, but many remain an integral part of the national health system, with close affiliation to the public sector. In most cases, the foundation of this association was on the basis of the FBNPs providing services to the poor and marginalised and reaching where the public sector and government could not [13]; with the focus of FBNPs being on addressing geographic access and the provision of health services for all, even before the concept of UHC was popularised [14, 15]. Within SSA, the relationship between FBNPs and their national governments has fluctuated in strength and enthusiasm over the last decades, influenced by individuals, national developments, and changeable global economic-, development- and health strategies - all impacting on thinking about the appropriate level of private sector engagement towards public health goals [10–13].

Despite the longstanding role of FBNP providers, their relationship with the government and their common integration into the national health system, there is a distinct lack of robust evidence on their contribution, historical development, relationship with the public sector, and their contribution to UHC and to the strengthening of whole national systems [16, 17]. The research that has been conducted to test the validity of the suppositions around the impact of FBNPs has highlighted missing information gaps and resulted in further unanswered questions [16, 18]. This is particularly pertinent in the context of increased urbanisation and changing population dynamics occurring across SSA. In relation to FBNPs working towards UHC, a lack of empirical evidence – in particular on their geographical presence - has led to repeated calls for the ‘mapping’ of religious health assets [17, 18]. Frequently excluded from provider registers, it has been argued that mapping is required to understand their contribution and to ‘put them on the map’ [19, 20], to increase the empirical evidence base [21] and to enable strategic engagement with partners [22]. These calls for mapping continue, with the most recent coming from the World Council of Churches (WCC), which in late 2017 called for a renewed international programme to map Christian health services in Africa [23]. In this article we report on a study which mapped FBNP health facilities in Ghana, utilising geospatial mapping technology to explore FBNPs contribution to UHC over time, focusing on geographic access.

### Country context: the Ghanaian health system

The Ghanaian health system architecture reflects historical changes within the country and provides important contextual background to this research. Since achieving independence from colonial Britain in 1957, Ghana has experienced significant political, economic and social upheaval. The population has increased from 6.7 million in 1960 to 24.7 million in 2010, with a rapid increase in urbanisation [24, 25]. Since 2005, economic growth has averaged over 7% a year, with middle-income status achieved in 2010 [26]. Although this rapid growth and urban transition has been accompanied by poverty reduction and improved infrastructure, it has also been accompanied by an increase in inequality [26]. Significant discrepancies in wealth are evident between the largely rural North and the urban South, which are also differentiated along religious, cultural and topographical lines. These transitions have inevitably been accompanied by significant changes in the health outcomes of the population and an epidemiological transition towards a double burden of disease, with a rise in the incidence of non-communicable diseases alongside existing and persistent infectious diseases, resulting in complex public health challenges [24, 27, 28].

The period preceding the achievement of political and economic stability (1957–1992) was characterised by frequent political regime change and instability, impacting on the functioning and composition of the entire public sector. Despite subsequent political changes, the current structure of the public health system (which itself has its origins in the colonial health system) is largely reflective of the changes that were made following the democratic elections of 1992 [29]. The Ministry of Health (MOH) has strategic oversight and financial responsibility for policy development. The Ghana Health Service (GHS), created in 1996, acts as an (semi) autonomous agency under the MOH, responsible for policy implementation and as the main provider of health services. The public sector is supplemented by a substantial proportion of NSPs (private for-profits and private not-for-profits). Despite the progress and development within the country, the health system has been described as inefficient and inequitable, with ongoing financing and human resource crises [24, 27, 30–32].

#### Towards UHC

The global movement towards the achievement of UHC has been sporadic and uneven but has been on the national Ghanaian health radar and political agenda even prior to it gaining prominence on the global stage, and is a key driver of health policy and strategy within Ghana [32]. Driven by socialist ideals, the provision of free health services to all was a high priority of the post-independence Nkrumah government and its aspirational welfare state [29, 33]. However, this aspiration and attainability was lost in the following period of political instability and economic decline (1966–1981). The incoming Rawlings government, faced with this domestic challenge and both influenced and handicapped by the global policy of structural adjustment and ‘rolling back the state’, reduced spending on health and in 1985 re-introduced user fees (‘cash and carry’), negatively impacting on access to and utilisation of services and the health of the population [29, 34].^3^ However, since the achievement of stability with the democratic elections in 1992 and the subsequent afore-mentioned restructuring of the health system, two concerted and complementary efforts have been made towards achieving UHC and specifically increasing access to healthcare [31].

To address the issue of financial coverage, the government introduced the National Health Insurance Scheme (NHIS) in 2004. This was on the back of an election promise, made in light of increasing public resistance to the ‘cash and carry’ system and in line with the international focus on the necessity of mandatory pre-payment mechanisms in order to achieve UHC [35]. We will not repeat the heated debate on the NHIS in the broader literature [31, 36], which has been both applauded (for being innovative, progressive and pro-poor) and denounced (for not achieving these objectives [34, 37]), and is beset by significant financial and operational difficulties [36].

In order to address the geographic service inequities within the country, the government has rolled-out the Community-Based Health Planning and Service (CHPS) programme. This is specifically intended to increase access to services in remote and underserved areas, reorienting focus towards primary health care (PHC) in the community [38]. CHPS zones were created, with some having a physical structure (CHPS compounds) for the provision of services and staffed by salaried community health nurses, while other zones had no such physical structure. Following successful pilot studies in the Upper East region in the 1990s, the programme was rolled-out nationally (from 2005) in order to address the needs of marginalised communities [39], with rapid expansion in the last decade. Despite this effort, physical accessibility and geographic inequity remain a major concern [40].

### The role of FBNPs in Ghana

The FBNP sector in Ghana, as in many other SSA countries, has largely evolved from colonial-era Christian missionary origins. Today, this sector consists of a number of providers of varying religious backgrounds including faith-oriented non-governmental organisations (NGOs), non-networked facilities, informal faith-based initiatives and the Ahmadiyya Muslim Mission (AMM), amongst others. However, it is historically and currently dominated by mainstream Christian health facilities networked under the umbrella of the Christian Health Association of Ghana (CHAG) [41].

Similar to the other 22 Christian Health Associations (CHAs) present across SSA, CHAG is a network of local mainstream Christian member denominations that own health facilities [42]. Established in 1967, in a period of national upheaval, the original objectives set out by the founding members aimed to improve service coordination and to act as a consolidated platform for dialogue with the government [17, 42] - objectives which still apply today. It is understood that a key founding focus was to provide services to marginalised, rural and most in need communities although actual motivations are not clearly defined in the historical literature. However, CHAG has continued to maintain the ethos of the founding missionaries, with one of their current core values being to provide services to the poor and marginalised. CHAG has a long-standing, formalised and robust relationship with the government, solidified through the outcomes of the government’s Adibo Commission in 1975, working collaboratively but autonomously. It is a recognised agency of the MOH (in a similar but more limited fashion to GHS), with facilities receiving partial support and having signed a Memorandum of Understanding (MOU) with the MOH in 2003 detailing the relationship (2006 addendum) [41, 43]. FBNPs networked by CHAG are integrated with the public sector, submitting data to district health information software (DHIS) [44] and were put on the ‘fast track’ for NHIS accreditation. Due to this formalised integration and their long-standing role CHAG is considered one of the most robust CHAs in SSA [42]. Currently, CHAG reports that it has a membership of 282 facilities and 18 training institutions across all 10 regions of the country and constitutes 25 different ‘churches’ (Christian denominational groups). It remains a significant player in the Ghanaian health system and self-reports that its network provides 35–40% of the health care in the country with just 5.5% of the infrastructure [45]. (As with the other CHAs, these estimates of market share are contested [16, 41, 46, 47].)

The relationship between CHAG and the government has inevitably fluctuated since its formation. The afore-mentioned decades of national upheaval combined with shifting global economic and development trends and changing health policies have been influential both in the governance of the overall health system, as well as the rhetoric surrounding the involvement and role of FBNPs. In common with other FBNPs, CHAG has had to continually demonstrate its ‘value-add’ or contribution towards the achievement of public health goals in order to maintain its status and continue leveraging resources from the government (particularly in light of decreasing funding from international denominational religious bodies) [17, 18]. Table 1 provides some insight into the tangible health system interventions relating to FBNPs that have resulted from periods of strong collaboration (such as the financial subsidies from government following the Adibo Commission and the signing of the MOU). Although relations between CHAG and the government are described as being generally robust [42], tensions have been reported, most recently in relation to the significant reimbursement delays in the NHIS scheme [45]. This recent tension is exacerbated by CHAG’s need to continually demonstrate their value and negotiate their position, particularly in light of the changing population dynamics, increasing urbanisation in historically rural areas (where CHAG facilities are traditionally present), and the developing capacity of the government to meet the health needs of the population through the public sector. Strongly influenced by the historical and contextual factors that are examined in this article, it is noted that the dynamic between CHAG and the government is also driven by often-invisible personal relationships between actors [3] which are a challenge to assess.^4^

**Table 1:** Summary of Ghanaian historical context and key health system events

Alignment to Fig. 4	Year/Timeline	Socio-Political Context	Key Health System Events (as related to FBNPs)
Up to 1949	Up to 1844	Pre-colonial era	Little medical missionary work
	1844	Bond of 1844: Traditional chiefs sign Bond allowing British Government to rule	
	1844–1914	Period of strong British colonial administration. Colonial structure: Gold Coast colony, Ashanti (interior), Northern Territories Protectorate	Colonial health system and infrastructure established serving largely European populations in coastal (colonial) areas Significant increase in Christian missionary activities especially into the interior but largely focused on education and evangelism
	1914–1945	World War I, Great Depression and World War II	
1950–1969	1952	Kwame Nkrumah becomes Prime Minister but shares power with British governor	Maude Commission: Recommends support of the mission health sector Throughout the 1950s, significant growth in mission hospitals
	1956		Agreement around ‘agency’ hospitals – those run by missions but supported by government
	1957	Independence: Gold Coast becomes the independent state of Ghana	
	1960	Nkrumah becomes President (1960–1966) Period following independence characterised by massive political and economic instability	Nkrumah government has a strong focus on social development and welfare state
	1962		Maude Commission into policy
	1967		CHAG established
1970–1989	1975	Continued considerable economic and political instability.	Adibo Commission: Confirms importance of mission hospitals and recommends government pays salaries in CHAG facilities
	1978		PHC strategy formed (influenced by Alma Ata)
	1981	Jerry Rawlings takes power through a military coup	
	1985		User fees reintroduced
	1987–1989	Period of structural adjustment	
1990–2009	1992	Democratic constitution passed Rawlings wins election with National Democratic Congress (NDC) party	
	1996	NDC/Rawlings re-elected (presidency 1981–2001)	GHS established
	1999		CHPS launched
	2000	John Kufour of the New Patriotic Party (NPP) wins election (presidency 2001–2009)	
	2003		CHAG-MOH MOU signed
	2004		NHIS introduced CHAG received immediate NHIS accreditation
	2005		National roll-out of CHPS
	2006		Addendum to CHAG-MOH MOU
	2008	John Atta Mills of NDC wins election (presidency 2009–2012)	
2010 onwards	2010	Ghana classified as a middle income country	
	2011	John Mahama of NDC wins election (presidency 2012–2016)	
	2016	Nana Afuko-Addo of NPP wins election (presidency 2017-present)	

Source: author. This table provides a brief context of the political, social and economic history of Ghana as well as significant events in the development of the public-private health system. A detailed history and analysis of the development of the national health system is reported in a forthcoming article by Olivier and Kwamie (2018)

Today, CHAG seeks to maintain both the ethos and functions on which it was founded - but in a very different national health system context [45]. As the relationship with the government has evolved and as CHAG itself has grown to include more members, questions have been raised around its role, value-add and comparative advantage [16]. Some studies have examined aspects of FBNP contribution in Ghana [41, 48, 49] but these have tended to focus on one snapshot of time and on varied comparative aspects. There has been little historical focus on the evolution of the relationship between CHAG and the government and whether it did and still does contribute towards UHC and health systems strengthening.

## Methods

This study seeks to address the question: ‘How have faith-based non-profit providers contributed to the historical development of the Ghanaian health system towards UHC?’ – with a focus on geographic availability and distribution. Framed as Health Policy and Systems Research (HPSR) and utilising an associated interdisciplinary approach, a flexible mixed-methods strategy was conducted which drew on multiple forms of data and combining extensive synthesis of literature, documentary and archival research with geospatial mapping. The research was conducted in three phases during 2017, with reiterative data analysis, synthesis and triangulation throughout.

In Phase 1, a scoping literature review was undertaken focusing on three main areas; the historical development and current status of FBNPs in SSA, the contribution of FBNPs to UHC, and geospatial mapping of FBNPs in SSA. Only seven studies were found explicitly conducting research on *geospatial* mapping of health-focused FBNPs in SSA, reflecting the scarcity of literature on this issue. This review formed the foundation for the closer focus on Ghana in this article. Peer-reviewed and grey literature as well as secondary and archival documents were collated and thematically organised around the three mentioned focus areas (FBNPs in SSA, FBNPs contribution to UHC, geospatial mapping of FBNPs in SSA). A number of relevant organisational websites were also identified and searched including those maintained by CHAG and the GHS.

Phase 2 of this research involved geospatial mapping. This is a methodology originating in geography but one that is increasingly being used within public health. It remains under-utilised in resource-poor settings such as SSA [50, 51], partly due to methodological challenges including poor data access and quality, lack of technical capacity and the necessity to rely on hardware [52, 53]. However, it is becoming more feasible with the increased availability of open-source geographic information system (GIS) software. Much GIS mapping work in SSA (including Ghana) has tended to focus on disease trends [54], but visual representations of the health system can also provide insights into access, service provision and utilisation – especially for planning and management [55–59]. Despite the repeated calls for mapping of FBNP assets (outlined above), surprisingly little work has been carried out. There is a tendency for FBNP data to either be excluded from national data sets or subsumed as part of other public or private sector data. The limited work which has been done on FBNP providers, tends to focus on narrow geographical areas, particular service aspects, and on one point in time – always stressing the limited availability and quality of data. Only one directly relevant study has been carried out on FBNPs in Ghana (referred to in the results section) [49].

The data collection for this phase involved the gathering and synthesising of a variety of quantitative and qualitative data in order to build as accurate a master list of health facilities as possible. The foundational data source for building this master list was a geo-referenced list of public and private facilities (also including directorates, teaching and research institutions) which was gathered by and bought from the Centre for Remote Sensing and Geographic Information Services (CERSGIS) based at the University of Ghana. This was first collated in 2008 with periodic updates occurring until 2015. This CERSGIS database consisted of 2803 data points (we removed 21 clear duplicates). One hundred eleven items classified as health directorates were also removed from the database. This list was cross-checked and discrepancies reconciled with primary data collated in 2005 for the WHO Service Availability Mapping (SAM) report [60]^5^; an open-access resource of health facilities maintained by GHS [61]; and varied other documents. As the focus of this study was on FBNPs and with CHAG being the dominant service provider within this sector, the database was supplemented with a 2017 membership list from CHAG, consisting of 300 member facilities. One hundred seven of these 300 were not included or identifiable in the CERSGIS database. These facilities without GPS locations were geo-referenced manually by matching facility, town or village names on Google Maps or through Google searches and included in the master database. It should be noted that some of these are approximate, but considered adequate for the visualisation purposes of this research. It was not possible to geo-reference 14 of the CHAG facilities (data kept for non-mapping reference purposes but excluded from the master database). The final verified list consisted of 2795 data points composed of public and private (for- and not-for-profit) facilities, teaching and research institutions.

Due to the historical nature of this study (and the desire to show change in the health system over time), it was important to gather information on the establishment dates of FBNP facilities. Given the constraints of the research process, this could only include current CHAG members (that is, not inclusive of the small number of ‘other’ FBNP providers in Ghana).^6^ Data on date of facility establishment was acquired for 224 of the 300 members – with this information gathered from CHAG Head Office, the National Catholic Health Service (NCHS), and the Salvation Army’s national databases – and supplemented by internet, literature and (FBNP) archival searching. Substantial efforts were made to acquire missing establishment data as well as the date that facilities became a member of CHAG, although not entirely resolved.^7^ There were considerable discrepancies which were resolved or validated as far as possible through cross-checking during the final phase of the research (contextualisation). The acquisition of data on the establishment dates of public facilities were more challenging to gather and was not done systematically due to time constraints and as the primary focus of the research was on FBNPs.

The next stage of the mapping phase involved the creation of the maps. Data points were plotted using global positioning system (GPS) coordinates in a variety of presentations with appropriate base maps chosen and developed. QGIS™ software was used, chosen over other options such as ArcGIS™, because it is free and open-source and therefore more accessible to stakeholders (for future utilisation by local stakeholders). The advantage of GIS software (over for example Google Maps) is that multiple data sets can be combined and displayed as independent layers enabling simultaneous visual assessments of the data. Technical challenges and limitations are noted in the results section.

Phase 3 of the research involved the synthesis of primary materials in order to contextualise the maps. Visual trends and patterns were assessed against reported events and the digital maps were compared against historic paper-based maps. These materials included organisational annual reports, archival materials, paper-based maps, key informant transcripts acquired for the primary study in which this mapping study is embedded,^8^^,^^9^ and historical documents found in CHAG and NCHS archives during a research fieldwork trip in February 2017.

Processes to ensure and maintain rigour were applied throughout the research. This included multiple methods of design and data collection, a clear account of all steps taken throughout the process, constant data analysis and synthesis, triangulation (cross-verification) of all data and findings, and peer debriefing and support [4]. Establishment and membership data were further verified by CHAG, NCHS and the Salvation Army. The researchers conducting the primary study from which some data were drawn also took appropriate steps to maintain rigour, including cross team analysis and member checking (participant validation) of interview transcripts.

Ethical standards were maintained throughout the research, complying with international, national and institutional requirements. Although considered minimal risk, ethical approval was obtained from the University of Cape Town’s (UCT) Human Research Ethics Committee (HREC) (Reference 303/2017). The study also operated under the existing ethical considerations of the primary study, approval for which has been obtained from the WHO Research Ethics Committee, GHS Ethical Review Committee and UCT HREC (annually updated, 2015–2018). In addition, although not a formal requirement, permission was obtained from GHS, Ghana Department of Health and CHAG for this sub-study.

## Results

In this results section, we have merged primary geospatial mapping results with integrated secondary literature, as the most appropriate way to express the contextualised maps.

### The public-private health system today

**Fig. 1:**
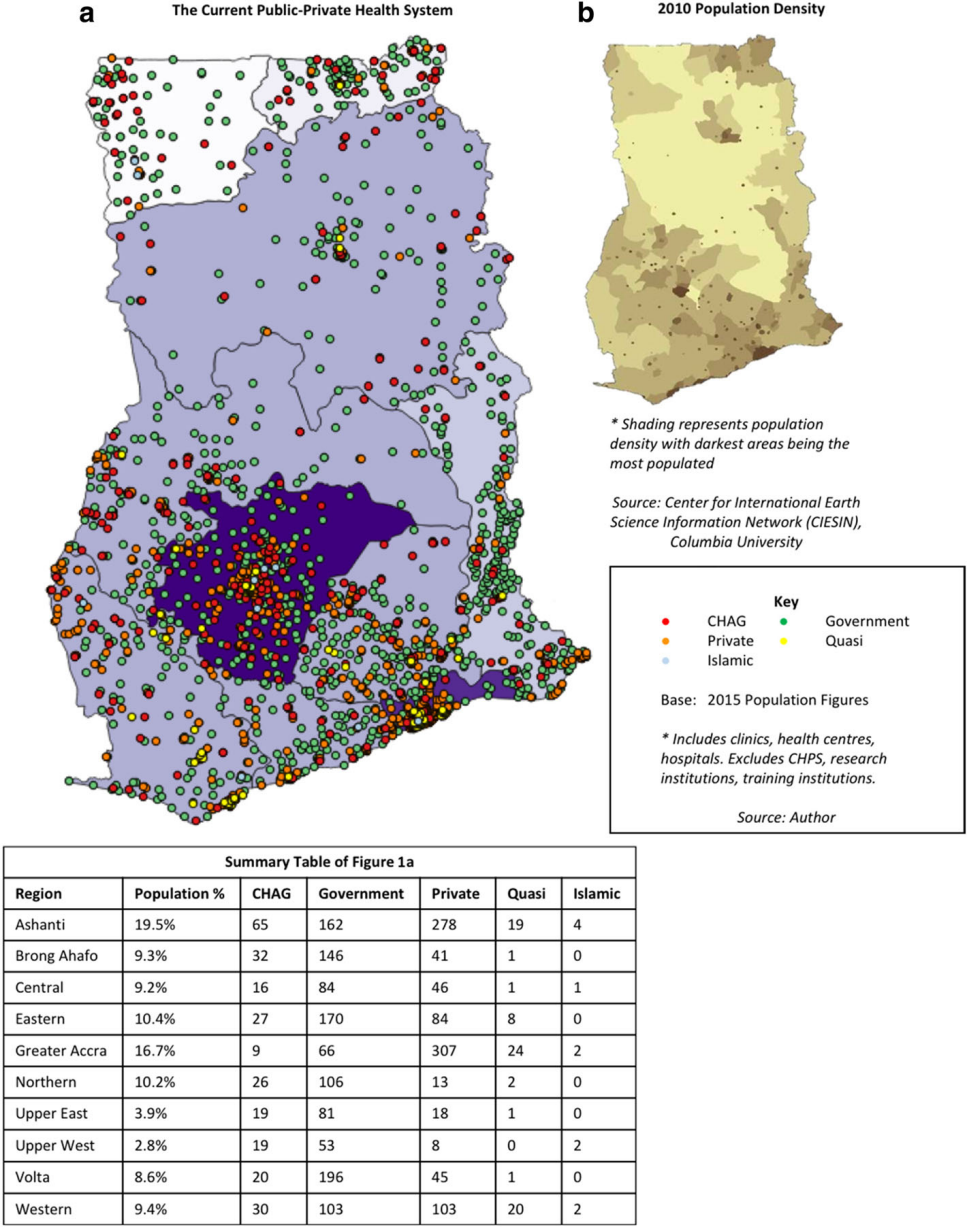
**a** The Current Public-Private Health System **b** 2010 Population Density

The current distribution of all health facilities in Ghana continues to be geographically inequitable, as represented in Fig. 1a. This map is representative of the health system in 2015 with CHAG details as of 2017.^10^

**Fig. 2:**
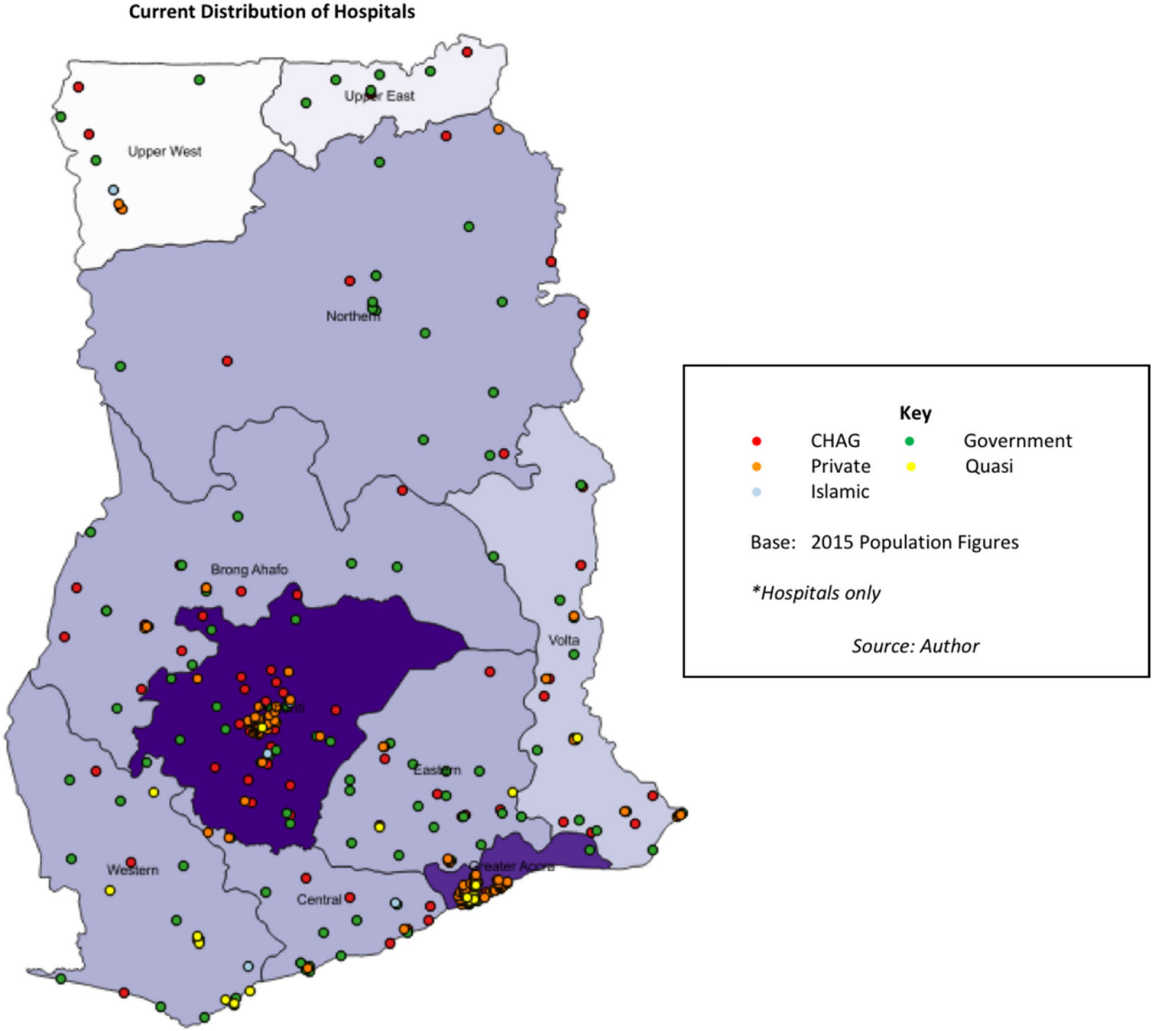
Map of current distribution

CHAG and government facilities currently have similar distribution patterns across the country and these largely correlate with regional population density (further detailed in Fig. 1b). Although the population is lower in the Northern and Upper regions, it is clear that people who are living in those areas remain underserved and need to travel considerable distances to access health care. Combined with the lack of infrastructure and transport systems typical in rural areas, this is likely to be a disincentive to accessing and utilising care. This visual representation corresponds with literature which states that unequal geographical distribution and regional disparities remain a significant issue in Ghana [24, 40]. It also reinforces the findings of the 2007 SAM report that health facilities (and associated human resources and infrastructure such as piped water) are unequally distributed with Northern regions underserved [60].

Figure 1a also highlights that ‘other private’ (private for- and not-for-profit, but not FBNP) facilities are not evenly distributed. The CERSGIS database from which these figures were drawn does not differentiate between for- and not-for-profit providers in this category. However, one assessment of the Ghanaian health system (based on 2009 data), estimated that approximately half of all facilities are privately owned [24]. Within that, 73% are classified as private-for-profit (making up 36% of the total health system) with the remainder of the private category composed of quasi-public,^11^ CHAG and Islamic providers. Based on this, it is estimated that the majority of providers categorised as private in Fig. 1a are for-profit providers.

‘Other’ private providers are mostly concentrated in the cities and urban hubs [62], particularly in the Greater Accra region and around the city of Kumasi (Ashanti region). Although Kumasi is also served by a number of government and CHAG facilities, conversely, our analysis shows that currently just 3% of CHAG and 5% of government total national facilities are located in the Greater Accra region (supported by Oboudi’s findings of just three CHAG facilities in Greater Accra in 1999 [63]). This finding could be attributed to two characteristics of the Ghanaian historical health system context. Firstly, the low number of FBNPs in the Greater Accra region is likely representative of their historical placement and development, consciously not present in colonial areas such as Accra (discussed in the following section). Secondly, it potentially signifies the success of strategic government efforts to increase geographic access, reflecting concerted health infrastructure development away from the historically well-served (colonial administration) areas. However, the limited number of public sector and CHAG facilities in the Greater Accra region, does raise the question of what happens to the urban poor or those unable to pay for private health care in this densely populated region.

In assessing UHC, the level of care (from primary health centre to tertiary district hospital) must also be considered. Figure 2 shows that hospitals (higher-level facilities) are sparser in the Upper and Northern regions of the country. Private hospital providers are particularly predominant in the urban areas of Accra and Kumasi [64]. These findings reiterate the earlier observations about the whole health system (Fig. 1) – but there are noticeable differences, in that CHAG and government hospitals are mostly not located geographically adjacent to one another (apart from around Kumasi), and this is especially evident in the Northern and Upper regions. It should also be noted that many of these CHAG hospitals are ‘district designated’ (serving 100,000–200,000 people with 50–60 beds [65]) – so act as district or regional hospitals for the public sector system.^12^ (GHS provides some guidance on the functions, size and minimum criteria for different levels of facility within the health system [65, 66]).

### 1957: the health system at independence

**Fig. 3:**
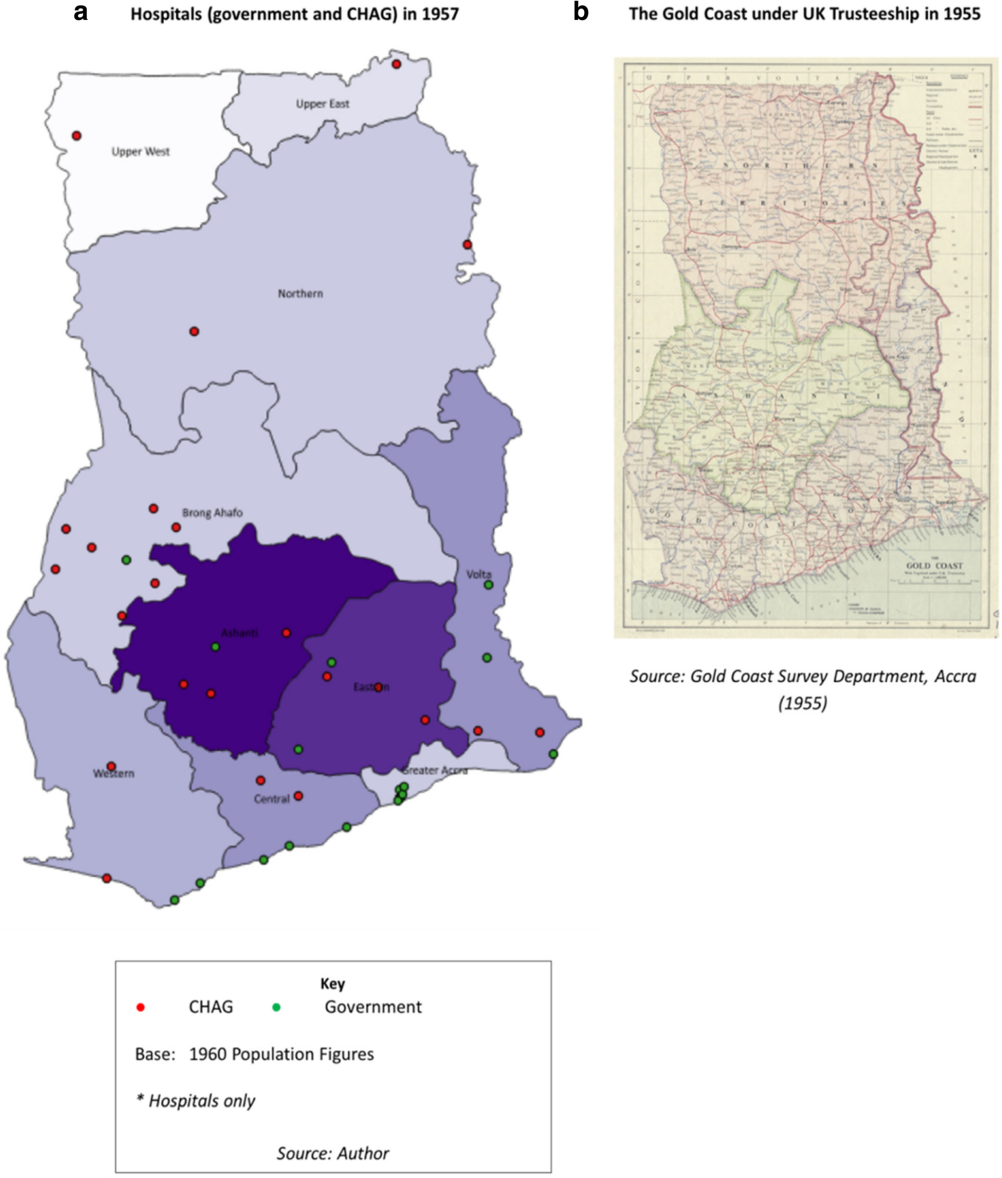
Hospitals in 1957. **a** Hospitals (government and CHAG) in 1957. **b** The Gold Coast under UK Trusteeship in 1955

Figure 3a shows the distribution of hospitals (only) established at the time of independence, up to and including the year 1957 against the 1960 regional populations (earliest available – drawn from the 1960 census data). Of the 23 FBNP hospitals plotted, 17 are Catholic, representative of early missionary denominations and their long-standing dominance within the FBNP sector [41]. Establishment data for government facilities was not systematically gathered but the data available for older government hospitals known to be present in 1957 were used. Classification is according to their current status in terms of ownership (CHAG versus government) and facility type (clinic versus hospital) and it is noted that some of these have naturally changed over time.^13^

The colonial period was marked by significant development in the Southern and Coastal regions (the Gold Coast), with little attention focused on the resource-poor northern territories (see Fig. 3b for colonial boundaries). This was reflected in a highly segregated health system serving the needs of the colony [29, 33]. These areas of concentrated socio-economic development have left a long-lasting legacy, still evident today [67], as shown in Fig. 1a. The literature suggests that missionary health work followed the geographic paths carved by earlier missionaries, who were more focused on proselytization and education, but who developed linked patterns of settlement (‘path dependency’ [68]). Mission facilities deliberately expanded beyond colonial administrative boundaries (as represented in Fig. 3b as the Gold Coast Colony), reaching out and providing services in rural and marginalised areas where government facilities were not present (20,160,905 CHAG, unpublished interview transcript) [45]. Although it was difficult to verify the location of government facilities at this point in time, Fig. 3a corroborates these findings, showing hospitals in the Northern and Upper regions as *only* operated by FBNPs (missions) and government hospitals mostly situated along the coastline, with some in Ashanti and one in Brong Ahafo region (20,160,907 retired MOH leadership, unpublished interview transcript).

### The evolution of FBNPs

**Fig. 4:**
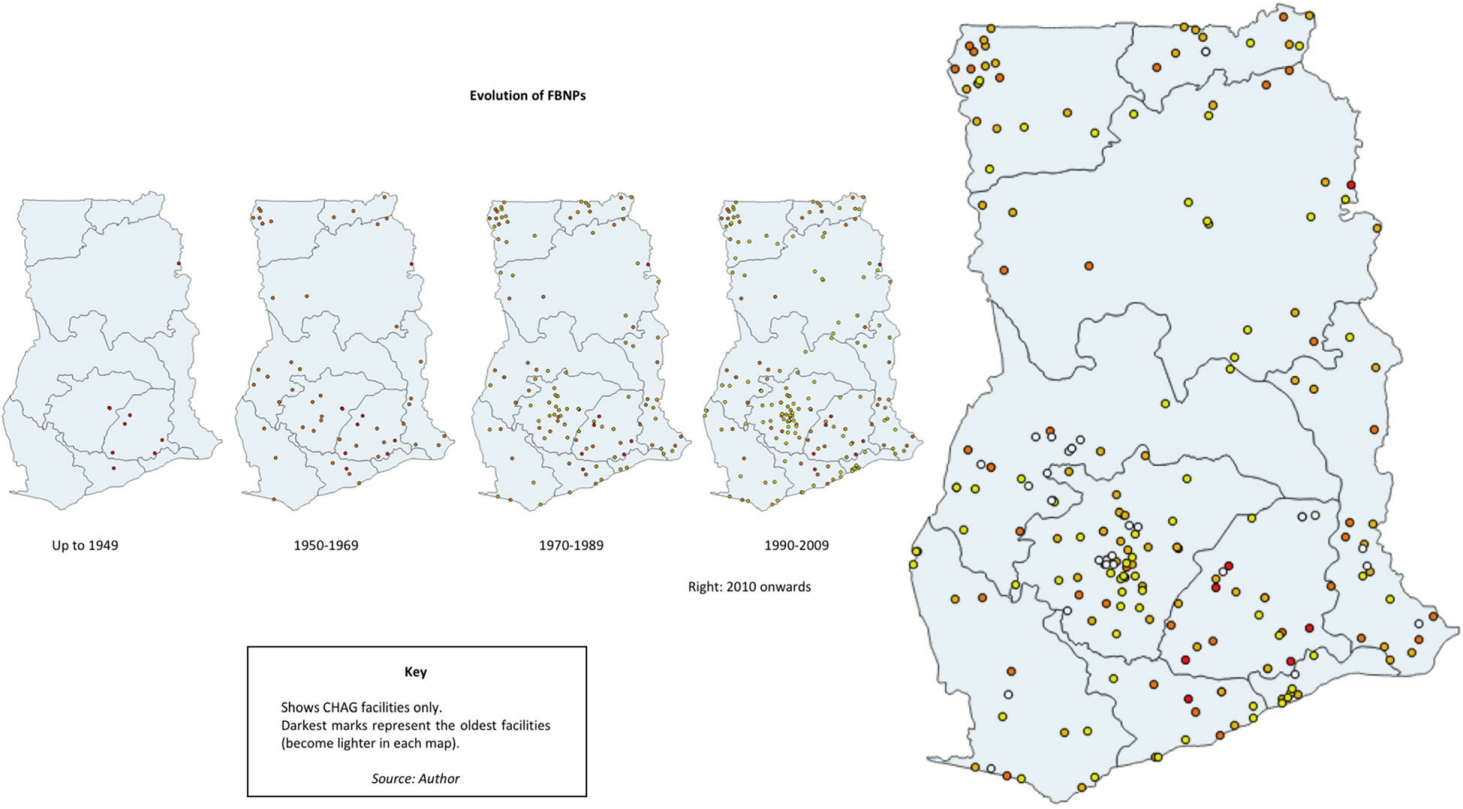
Evolution of FBNPs

Figure 4 provides a simple representation of the evolution of FBNPs over time, based on establishment data.^14^ This is based on current CHAG members and does not include other FBNPs that exist.

Secondary literature documents a rapid increase in the number of both government and missionary services in the immediate pre-independence period [29, 33]. This proliferation of FBNPs is supported by Fig. 4 with 31 FBNP facilities established between 1950 and 1959. Many of these were in areas further away from colonial boundaries. It is possible that it was associated with the 1952 Maude Commission which supported the enlistment of missions in the national provision of health [33]. Other possibilities include that it could have been as a result of the pre-independence economic upturn or positioning in preparation for independence, recognising the impending increased unification of the country. The latter half of this decade also marked the advent of ‘agency’ hospitals. These were largely located in rural areas, where construction was paid for by government, but otherwise owned and run by the churches [43, 69]. Examples include Bawku Presbyterian Hospital in the Upper East region (established 1955) and St Joseph’s Catholic Hospital in the Upper West region (converted from a clinic to a hospital in the 1950s).

The map also shows that there appears to be another significant increase in FBNP facilities during the 1980s (46 FBNPs established). This was an unstable period with tough conditions in the country (20,160,907 retired MOH leadership, unpublished interview transcript). It was the era of structural adjustment and health user fees were reintroduced in 1985 [34]. It has been suggested that in times of crisis and when the public system is weak (particularly in fragile and conflict states), that NSPs emerge and become more prevalent [70] and the increase during the period could be attributed to this trend. Globally, this period is affiliated with a focus on ‘rolling back the state’ [71] thereby promoting the private sector, as well as a surge in international development aid (to both government and non-state actors) which may have benefitted FBNP providers. Another suggestion posited here is that the increase could also be linked to the 1975 Adibo Commission. This government commission investigated the role of mission health services and marked the commencement of formal collaboration between FBNPs and the MOH, with facilities receiving partial support for salaries and supplies, and in turn filling gaps in the national system [41, 43].

### How has CHAG changed?

**Fig. 5:**
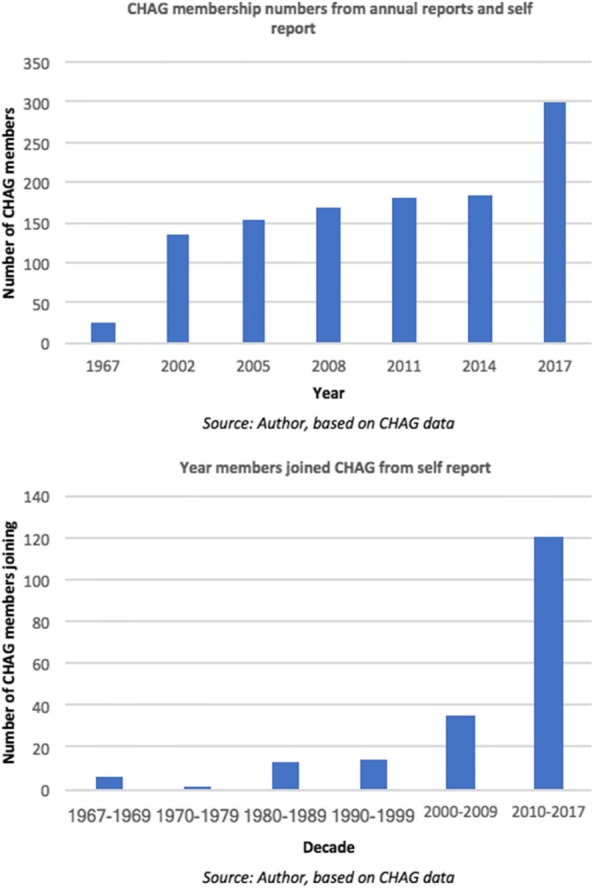
**a** CHAG membership numbers from annual reports and self report **b** Year members joined CHAG from self support

The FBNPs that are examined in Fig. 4 are all currently members of CHAG (2017), however, they have not always been. In some cases there are significant time lags (decades) between the establishment date of the facility and the date that they became a member of CHAG. This is applicable even for some of the facilities owned and run by the founding denominations (despite indications that they should have become CHAG members immediately), but there is no clear explanation from members for why this is the case and reflects data limitations. Therefore, it would have been useful to carry out the same temporal mapping exercise visualising and contextualising changes in CHAG membership. Unfortunately, membership data (the date they joined CHAG) were only available for 189 of the 300 CHAG member facilities and of those, 152 are recorded by CHAG as having joined since the year 2000 (displayed in Fig. 5). Due to this lack of information, it was not suitable for mapping analysis.

Despite this, an interesting trend is noted in the later data with a rapid increase in membership in 2015. CHAG state that they had a two-year membership freeze resulting in many facilities becoming members at the same time (in 2015) (CHAG, personal communication, 19th July 2017). However, even accounting for this, an increase of approximately 120 facilities on a base of 180 over this period is huge. It is not clear what the cause of this is - when the 120 are analysed, there are no clear trends in denomination or geography. It is also not related to a proliferation of new facilities as at least half of them were established prior to 2013, with a number dating back to the 1960s. It is suggested that it could relate to CHAG’s fast-track accreditation of the NHIS or conversely, increased sectoral solidarity in facing the financial reimbursement challenges around NHIS [45] - but these conclusions are not conclusive.

### Do CHAG serve the marginalised today?

**Fig. 6:**
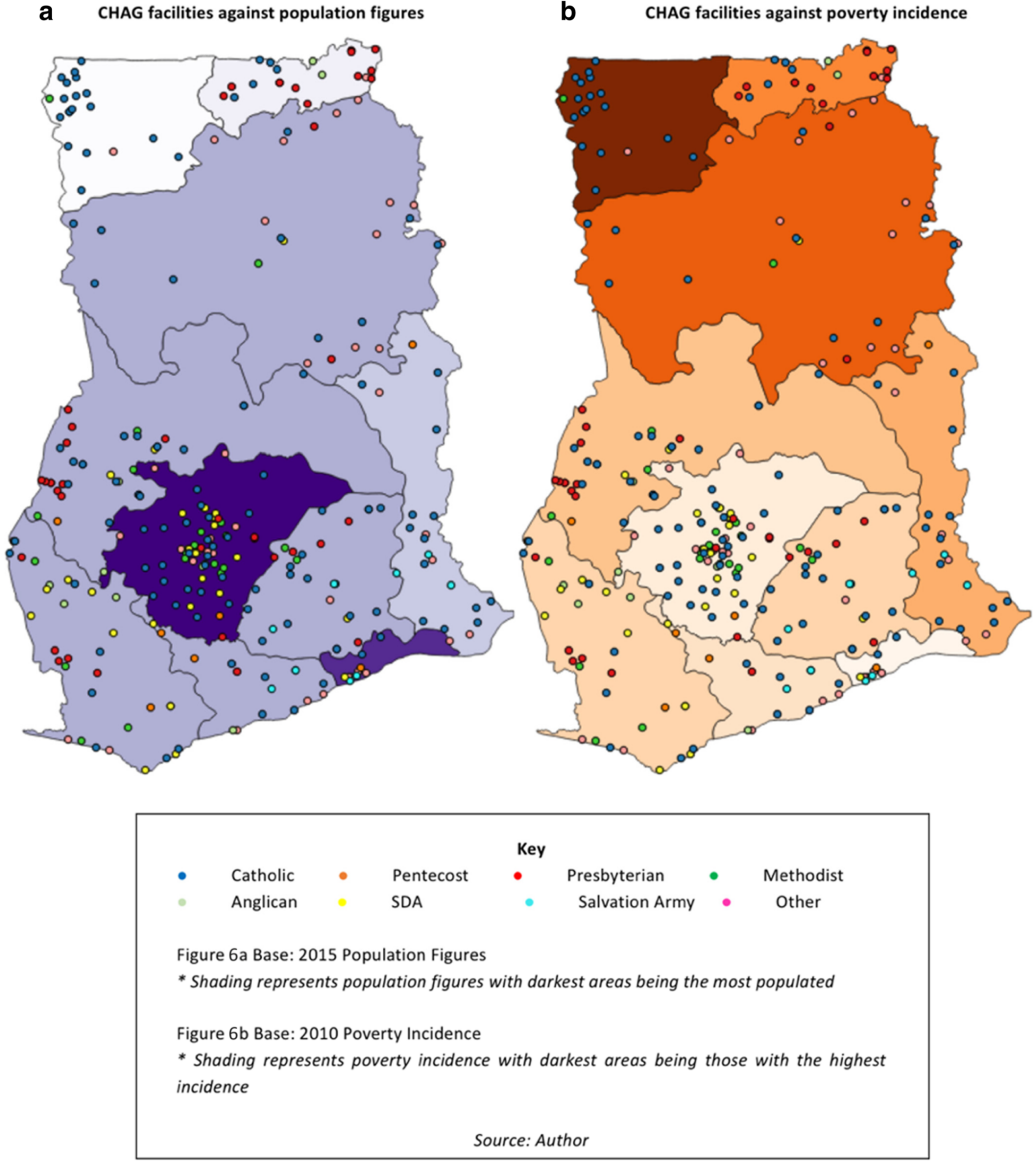
**a** CHAG facilities against population figures **b** CHAG facilities against poverty incidence

Figure 6 plots current CHAG facilities against recent population (Fig. 6a) and poverty (Fig. 6b) figures by region. Population distribution and poverty are linked - highly populated urban areas in Ghana have lower levels of poverty than more sparsely populated rural areas [26] and this is evident in the two base maps. These maps seek to further assess this dynamic in relation to CHAG facilities.

Figure 6a shows that if regional population numbers are taken as a measure of urbanisation, CHAG does not appear to serve more rural than urban populations. Although present in the Northern rural areas, their distribution, much like government facilities, is aligned with population figures (as shown in Fig. 1b). A recent article by CHAG (current and former Executive Directors) has acknowledged this rural-urban shift [45]. This finding is supported by Fig. 6b which plots the presence of CHAG facilities against regional poverty incidence (essentially the inverse of the population density). The map shows that there is no evidence that CHAG has significantly more facilities in more poverty-stricken areas than it does in more wealthy areas. These findings are further confirmed when examined at a district level (sub-regional), with CHAG not present in many poorer districts [49], such as Banda and Kintampo South in Brong Ahafo region, Sissala West in Upper West region and limited presence in the Gonja districts in Northern region (some of the poorest in the country) [25]. The government and CHAG both appear to serve (or not serve) the poor and rural areas to the same extent.

These findings are supported by the literature which has shown that CHAG facilities are primarily situated in urban areas [43] and when assessed on geo-location, do not disproportionately serve the poor [41]. In a mapping study, Coulombe and Wodon [49] found that CHAG facility locations (across all denominations) correspond more closely with the number of Catholics in the population rather than the degree of poverty, (although this itself has been influenced by the historical presence of missionaries - explored in more detail below). However, it is important to note that this study (and that conducted by Coulombe and Wodon) is examining poverty levels on a regional (or district) scale rather than at a household or individual level. This therefore limits the assumptions that can be made (as it does not track actual service provision against individual or household poverty status, but rather compares regional poverty levels and geo-location of facilities). However, the findings have been reinforced by statements in the key informant interviews which support the idea that CHAG is no longer in rural or poor areas and that CHAG’s focus should rather be on the quality of care that it provides rather than location (20,160,907 former CHAG, unpublished interview transcript; 20,160,907 retired MOH leadership, unpublished interview transcript). These findings suggest there are no conclusive answers on whether CHAG facilities serve the poorest individuals – at least not that can be found through this type of mapping study.

#### Denominational differences

When examining the distribution of CHAG, in addition to the temporal geographical representation, it is useful to assess the denominational split. As a founding member, CHAG has always been predominantly made up of Catholic facilities (through history, estimated between 40 and 60% of CHAG^15^), followed by Presbyterian facilities which are now the next largest denominational group [41]. Figure 6 shows that Catholics currently have the most facilities and the greatest reach (123 of the 300 member facilities or institutions). They have a strong presence in the Upper West region, an area historically neglected by the government, representative of their early mission work and considered likely resulting from expansion and outreach from the afore-mentioned agency hospital (St Joseph’s Catholic Hospital). The pattern is similar for Presbyterian facilities in the Upper East region (Bawku Presbyterian Hospital). Without contextualisation, this clustering could be seen as rural poverty being the priority of these organisations but is considered more likely that it is linked to the patterns of exploration and settlement (‘path dependency’) of early missionary work (although this itself is a circular argument as this early missionary work targeted rural populations). Another pattern that is evident from the maps is that the Seventh Day Adventists (SDA), with many recently established facilities, are largely predominant in urban areas (particularly around Kumasi) and have a minimal presence North of the Ashanti region. This shows important denominational differences even within the FBNP and CHAG clustering.

## Discussion

This research has focused on assessing the historical and present spatial development of FBNPs within the Ghanaian health system in relation to facilities provided by the government and other private sector, seeking to contribute important ‘baseline’ data to the growing body of research on the topic, and counter unsubstantiated claims around their contribution to health systems in SSA. Taking a systems perspective and viewing the health system holistically, the results demonstrate that there are areas of Ghana, particularly in the North, which despite concerted efforts to address geographical inequalities by both FBNP and the public sector, are still not having their (local) health needs met by either public or private providers due to a lack of facilities. A legacy of the development associated with the colonial period, this North-South divide has been a long-standing issue, but the health system must continue to be oriented to address it if Ghana hopes to improve access and achieve UHC by 2030 as outlined in the Sustainable Development Goals (SDGs).

As a particular type of NSP, FBNPs, largely coordinated under the umbrella of CHAG, have had a significant long-standing role in seeking to address this fundamental aspect of UHC. The historical analysis clearly shows that, in line with the work of the early missionaries and the founding focus of CHAG (framed as a founding mission but not clearly defined in the historical literature), they *did* originally spatially complement the government system, providing services in rural underserved areas and contributing towards improved geographic access. However, as the national health system has developed - with the geographical expansion of the public sector, FBNP funding patterns shifting away from traditional sources, and increasing urbanisation around the historically rural areas where mission facilities were located - this dynamic is now less apparent. Examination of the current location of government infrastructure demonstrates that these facilities are now also located in the more rural areas of the country and in some cases, appear to have been established where CHAG member facilities are also present (suggesting worrying duplication of services, rather than intersectoral service development). It is recognised that the health system is strongly oriented towards the public sector and conversely, it could be viewed that the establishment of government facilities in these areas once predominantly served by CHAG, is in fact complementary and supplementary to CHAG (rather than the reverse), contributing to strengthening the overall system.

In making this claim and in order to assess its validity, it is important to examine the health system more closely. When assessing the distribution of different levels of care, the spatial positioning of government and CHAG hospitals (higher-level facilities) reflects less overlap, with hospitals generally not situated within the same immediate geographic area (other than around the city of Kumasi). Many of these hospitals have been present for decades demonstrating considerable resilience and suggesting some degree of coordination between sectors and providers. Although the literature alone does not indicate definitively when or exactly how this occurs, the 1975 Adibo Commission report and the more recent 2003 MOH-CHAG MOU are considered to be structured platforms which facilitate this engagement. This collaboration is also highlighted by the fact that a number of CHAG hospitals are district and regional designated. This suggests that they may play an important governance role as the ‘hub’ for the local or district health system (an extremely important area for future research). Although the afore-mentioned pattern of geographic inequities also exist around this tertiary level of care, this analysis demonstrates that FBNPs continue to contribute to improved geographic access to higher levels of care (and therefore UHC), and that the initial assumptions of the findings may not be so clear cut.

At the other end of the spectrum, this mapping exercise has not visualised or included the distribution of CHPS zones (or compounds). However, given CHAG’s historical focus on improved geographic access for rural and marginalised communities and as CHPS is the government strategy to address this issue at a primary level, it is important to mention it here. Although not considered a duplication of services due to its small-scale community focus (often with no associated health infrastructure), the increasing national coverage of the programme means that it is inevitably present in areas where CHAG member facilities exist. Having been largely excluded from early roll-out of the programme, CHAG are only now beginning to coordinate with the government on CHPS [45]. In order to enhance the effectiveness of the programme (and because primary level programmes inevitably develop into secondary- and tertiary-level facilities), intersectoral collaboration and improved systems integration seems something to be encouraged.

In examining the evolution of FBNP providers in Ghana, it is worth considering the impact of the overall FBNP presence. It has been posited that those once-rural areas where facilities were located, proceeded to urbanise and develop – often *because* of the presence of Christian churches and communities and their associated health and education services (and presumably transport links), thereby attracting people and ultimately government services, which resulted in more rapid development in these areas [45]. As one interviewee stated “Battor Catholic Hospital made up what Battor is, so CHAG is the community” (20,160,906/7 MOH, interview transcript). Alternatively (and to a lesser degree), it has been suggested that missionaries located themselves in areas where there were already local populations or which had favourable conditions (for example, situated at high altitude away from malaria) and as such were always likely to be areas of growth, regardless of their presence. There is no clear ‘cause and effect’ answer, but it is likely that missionary presence had an impact on the communities around them and in turn, have had a role in shaping the current local health system. This trend of infrastructure and community development around historic missionary locations is particularly noticeable in the denominational breakdown with clusters of smaller facilities present around some of the earliest and long-standing FBNP hospitals.

As with many SSA countries, the configuration of the population in Ghana has shifted and the results indicate that the infrastructure of the public health system has broadly adjusted to align with the current population distribution. However, one clear exception to this rule is the Greater Accra region where both CHAG and the government continued to have relatively limited presence. For FBNPs, this limited presence is likely a result of the aforementioned deliberate expansion beyond colonial boundaries – and the (earlier) strong orientation towards the ‘rural poor’. The reason for the paucity of government services is less clear, but may be a result of strategic government efforts to increase access to health services across other areas of the country, and the presence of several large quasi-governmental facilities in Greater Accra. Rapid in-migration to this region (the second most populated) has resulted in unsystematic expansion since the 1980s [24, 72]. This has brought with it an increasing urban poor with limited access to public health facilities but who at the same time are particularly susceptible to the associated environmental health concerns of informal settlements such as poor water and sanitation facilities (as demonstrated by having the most cases in the 2014–2015 cholera outbreak [73]). The finer details of this urban analysis are outside the scope of this study. However, provision of services to meet the needs of the urban poor in this region, could be a potential area of future collaboration between CHAG and the government – however, this may need to take a different form and approach to previous collaborations, and require different ways of thinking about intersectoral collaboration for whole systems development. There may also be denominational differences to overcome - as highlighted in the maps, the few CHAG members currently present in this area are mostly the smaller denominations sometimes operating just one facility in the country, highlighting the necessity for CHAG’s coordinating role to promote effective coverage *between* members. In addition, some of the newer member denominations such as the SDAs, have focused their expansion in urban areas, mostly around Kumasi, and may have potential to do the same in Greater Accra to meet the needs of the burgeoning urban poor.

The CHAG Secretariat have had a notably strong, long-standing collaborative relationship with the MOH [42], engaging effectively through successive governments, weathering periods of significant national turmoil and health system shocks. It is recognised that these findings on FBNPs geographic coverage may influence perceptions of CHAG’s role but these findings do not negate the value of their existing and potential contribution towards UHC or role in the national health system. Drawing on global recommendations around the importance of involving NSPs in health systems strengthening efforts, and given their integration within the system, the government should continue to capitalise on and leverage CHAG’s existing strengths and infrastructure. As the epidemiological transition towards non-communicable diseases occurs in the country and in considering the service package required to achieve UHC, other areas that CHAG have pockets of specialisation in such as orthopaedics, eye care, and mental health services could also be drawn upon. Furthermore, their congregational linkages into communities could be leveraged to enhance community care and increase access to those who remain underserved (so also has potential for the CHPS programme). Viewing the health system as a whole (through such geospatial maps), and continued collaboration through coordinated policy processes would avoid duplication and fragmentation of services – and therefore maximise geographic access the furthest extent of the existing (and developing) infrastructure.

More broadly, this research has demonstrated that the historical social, political and economic context of a country has a significant impact on the health system of today. Changing relationships between public-private partners significantly impact on the provision and spatial footprint of facilities. From an HPSR methodological perspective, this use of GIS software has demonstrated the wider applicability of geospatial mapping technology – especially when integrated with other forms of data. When contextualised appropriately (a key feature of the HPSR field), maps can suggest important lessons about the patterns of historical health systems development, so that future change can be anticipated – and also expanding GIS mapping beyond its traditional uses. Furthermore, these mapping techniques enable visualisation of multiple data sets in a variety of formats which can be adapted according to stakeholder needs – and can be a powerful tool for enhancing intersectoral stakeholder engagement. In the context of the FBNPs this can provide valuable insights into the distribution of their network members which traditional data formats cannot. On a national level, especially if updated, and if the private sector is categorised appropriately, it is a useful tool for analysis of the complex web of providers which make up ‘the health system’, with potential to assist decision-making and enrich health systems analysis.

### Study limitations and challenges

The limitations of this study are in two key areas – challenges around the availability and accuracy of data, and limitations in terms of the scope of the research. The mixed methods approach, involving the synthesis of a wide range of data sources, allowed for more rounded findings and triangulation – but also resulted in some data discrepancies. Cleaning and verification to resolve these issues are an expected part of the research process and although not a limitation, are noted as a challenge. Relatedly, although the use of secondary data sources for geospatial mapping is appropriate for this kind of small-scale study, the quality of the mapping relied on the accuracy and availability of the data. Adequate for the purposes of this historically-focused descriptive research, it emphasises the benefits of such data being open-source and the importance of keeping databases current.

As described in the results, this research highlighted fundamental data gaps, impacting on the comprehensiveness of the analysis. If available, these would not only contribute to the research but more importantly would help to define CHAG’s composition and contribution and ability to reassess their position within the health system. This reflects the importance of data if FBNPs are to remain viable and relevant in an ever more technological and results-driven environment, and in the context of national governments increasingly utilising these approaches. This was poignantly acknowledged by one participant in the research stating, “we know that we are contributing, even if we can’t show it […] and we need to document our activities better” (20,160,909 CHAG board member, unpublished interview transcript).

An additional limitation was the continued challenges to gather complete and reliable data on facility establishment dates (FBNP, public and other) – which would have enabled comparative tracking of service establishment patterns over time, which could then be correlated against health system and contextual changes noted at those times. Although efforts were made to gather such historical data (for example through internet and literature searching, and direct requests to stakeholders), this data remained uneven and somewhat unreliable. Such data, if complete, would enhance the historical analysis of FBNP’s contribution to UHC and is therefore recommended for future consideration in order to build on this research.

The focus of this study was on the spatial distribution of FBNPs. As noted in the results, there are limitations around this geospatial approach when assessing factors such as poverty and assessing the nuances of the individual and household population that facilities serve, such as catchment areas. Furthermore, it is recognised that a limitation of this focus on spatial distribution is that other aspects of UHC were not considered in detail. However, the baseline established (and similar methodology) could be utilised for further exploration in the context of FBNPs to assess geographical placement in combination with service quality, health outcomes, facility coverage areas, human resources, fiscal aspects, service package and considering specialisations that CHAG are known for they are known for such as orthopaedic and eye care [43]. moving the discussion beyond the historical focus on geographical distribution towards other areas of potential comparative advantage.

Lastly, it is noted that this research focused on mainstream and formal Christian FBNPs in Ghana (grouped under CHAG), due to their dominance and the availability of data. The cost-effective methodology used in this research could be further enhanced by combining it with other forms of mapping, such as participatory GIS, in order to examine providers of other faiths (in Ghana, Islamic), informal networks and in contexts outside of SSA. Research gaps and questions remain in these areas and could benefit from multi-source mapping approaches to build the evidence and knowledge base where literature is particularly sparse.

## Conclusions

This research demonstrates the decades-long presence, endurance (resilience) and adaptability of FBNPs through periods of significant national upheaval in the Ghanaian national health system - and focuses on just one aspect of their potential contribution towards the achievement of UHC (geographic access). It establishes a foundation and substantiates (where previous data and empirical evidence were lacking), their historical and continuing role in the provision of services. The findings on the evolution of CHAG members and their stimulus for joining is not conclusive but as indicated in the results there are clear spikes indicating periods of strength and suggesting that member institutions seemingly joining CHAG when they saw an increase in their political and strategic value in relation to the government or at times of crisis. Although context specific, FBNPs across SSA are considered to have followed similar paths of development and as such, patterns may be drawn. However, as emphasised throughout this research, in order to substantiate their role and demonstrate their continued relevance in the provision of health services, it is essential that FBNPs maintain (more) substantive data records than are currently available (publicly or within FBNP networks).

The historical analysis demonstrates how the past has an impact on the spatial footprint of the present-day health system (supporting the argument commonly found within HPSR, that a historical lens is critically important for understanding health system development). For example, the history of political and population change, topography, economic fluctuations and concentrated areas of infrastructural expansion, all impact on the development of the health system. The centrality of people and the changing relationship and power dynamics between actors and sectors are also significant. Addressing the legacy (and potential inequities) of the past requires a sustained, concerted and coordinated effort across all providers in the health system. Better approaches are needing for analysing whole LMIC health systems that take multiple types of providers into account (and these might look very different to high-income health system analysis because of the highly diverse actors and power dynamics). Across countries, recognising and addressing the impact of these broader contextual factors over time, as well as the (sometimes unintended) consequences of historical health systems strategies and decisions, would provide valuable lessons for current day systems management and future planning.

This historical research therefore highlights that ‘the health system’ in Ghana is not just the public sector – and since inception has comprised a mixture of public and private providers, a situation that is unlikely to change. Although the ultimate responsibility of addressing inequities and improving the health outcomes of the population is considered to remain with the state – this is a reminder that other types of providers might also consider this a primary goal and responsibility. Engaging with the full range of existing NSPs is necessary to build a strong and effective health system. A collaborative approach towards health systems planning and management, between the government and NSPs, utilising accessible open-source data would enable greater understanding of the full range and scope of services within the country. The generation and use of geospatial maps to visualise such data is one tool for identifying gaps and duplications in order to reduce fragmentation and maximise resources, enhancing this kind of holistic health system analysis. To supplement and add depth to this macro level mapping approach, a meso facility level assessment of service users is required to assess provision of services to the poor.

A resilient health system is one which can provide consistent everyday health services and withstand complex emergency situations – over decades. Achieving UHC means ensuring that quality effective health services are both financially and physically accessible for all - and as such are equipped to deal with current (past), emerging, and future stressors and shocks. A *resilient UHC-oriented national health system* is complex and multi-levelled – and this research suggests that it is more likely to be achieved if a *whole* systems perspective is taken, that is inclusive of and capitalises on the strengths and resources that continue to be offered by FBNPs and other NSPs in many LMICs.

## Abbreviations

AMM: Ahmadiyya Muslim Mission
CERSGIS: Centre for Remote Sensing and Geographic Information Services
CHA: Christian Health Association
CHAG: Christian Health Association of Ghana
CHPS: Community-based Health Planning and Services
DHIS: District health information software
FBNP: Faith-based non-profit
GHS: Ghana Health Service
GIS: Geographical information systems
GPS: Global positioning system
HPSR: Health Policy and Systems Research
HREC: Human Research Ethics Committee
LMIC: Low- and middle-income country
MOH: Ministry of Health
MOU: Memorandum of Understanding
NCHS: National Catholic Health Service
NGO: Non-governmental organisation
NHIS: National Health Insurance Scheme
NSP: Non-state provider
PHC: Primary health care
PNFP: Private not-for-profit
SAM: Service Availability Mapping
SDA: Seventh Day Adventists
SDG: Sustainable Development Goal
SSA: Sub-Saharan Africa
UHC: Universal health coverage
WCC: World Council of Churches
WHO: World Health Organisation

## Footnotes

1 NSPs are those that operate outside the direct control or oversight of the government and include both for- and non-profit providers.

2 The classification of a FBNP is one in which there is terminological discord. In this article, the term FBNP refers to non-state, non-profit health providers who self-identify as being driven by religious values.

3 Detailed histories can be found in Arhinful [33] and Bohmig [29].

4 Beyond the remit of this paper, this is examined more closely in other papers reporting on this study.

5 The geospatial data collated for the SAM report is intended to be publicly accessible but was not – and was provided directly by the Department of Information, Evidence and Research, at the WHO in Geneva.

6 Information on the Ahmadiyya Muslim Mission healthcare is scarce and little reliable or substantial data was found to be available on the establishment, location or numbers of facilities within Ghana however, the information that was available from CERSGIS was included in the final database for mapping purposes.

7 Multiple attempts were made to acquire the missing CHAG facility establishment data by comparing historical member lists, internet searches, and direct contact – however too many dates remained unconfirmed for a reliable analysis to be conducted on the full database. Information was also collated on the date that facilities became a member of CHAG (which is different to date of establishment), but the data was assessed to be too incomplete to report here with confidence (this will be pursued later). Information was obtained from CHAG for 189 of the facilities, but as 152 of these were listed as joining between 2000 and 2017 there was a time bias in the data that could not be reconciled.

8 This research project is a sub-study within a broader WHO - Alliance for Health Policy and Systems Research (AHPSR) funded project conducted by researchers at the University of Cape Town (UCT) and the University of Ghana examining the historical relationship between the Ghana public health system and non-state non-profit providers.

9 Member checking was conducted as a form of verification within the main study. Furthermore, within this sub-study, gathered and synthesised data was again checked with key stakeholders.

10 Applicable across all of the maps, regional and district boundaries (now increased to 216) have changed over time but by using GPS data rather than address details this is not considered to be a significant issue for this research.

11 Quasi-public facilities are institutions owned by government establishments in service industries such as mining and manufacturing as well as educational, military and law enforcement agencies. They are subsidised but not operated by the government. They predominantly serve their target populations but use has been encouraged in times of crisis such as during physician strikes.

12 District designated hospitals are the first referral hospital and provide higher levels of clinic care at the district level. They serve populations of approximately 100,000–200,000 people in a clearly defined geographic area [65].

13 This point is applicable across all of the maps and is a recognised limitation of the study with challenges in showing changes in ownership and type of facility, as well as the exclusion of facilities that have subsequently closed.

14 Establishment data is available for 224 of the 300 CHAG member facilities.

15 This estimate is based on limited figures due to the previously mentioned lack of CHAG membership data available. A 2003 CHAG Annual Report shows that 67.5% of total facilities and training institutions were Catholic and the latest figures show that they make up 41% of CHAG members.
